# Optimal Revascularization Timing of Coronary Artery Bypass Grafting in Acute Myocardial Infarction

**DOI:** 10.1002/clc.24325

**Published:** 2024-08-14

**Authors:** Hyo‐Hyun Kim, Myeongjee Lee, Kyung‐Jong Yoo

**Affiliations:** ^1^ Division of Cardiovascular Surgery Ilsan Hospital Go‐Yang South Korea; ^2^ Biostatistics Collaboration Unit, Department of Biomedical Systems Informatics Yonsei University College of Medicine Seoul South Korea; ^3^ Division of Cardiovascular Surgery, Severance Cardiovascular Hospital Yonsei University College of Medicine, Yonsei University Health System Seodaemun‐gu Seoul South Korea

**Keywords:** acute myocardial infarction, coronary artery bypass grafting, National Health Insurance Service, postoperative outcomes, revascularization timing

## Abstract

**Introduction:**

Acute myocardial infarction (AMI) is a major global health concern. However, the optimum timing of coronary artery bypass grafting (CABG) in AMI patients remains controversial. This study investigated the optimal timing of CABG and its impact on postoperative outcomes. We hypothesized that determining the optimal timing of CABG could positively impact postoperative outcomes.

**Methods:**

We conducted a nationwide retrospective analysis of the National Health Insurance Service of Korea database, focusing on 1 705 843 adult AMI patients diagnosed between 2007 and 2018 who underwent CABG within 1 year of diagnosis. Patients were categorized based on CABG timing. Primary endpoints included cohort identification and the time interval from AMI diagnosis to CABG. Secondary endpoints encompassed major adverse cardiac and cerebrovascular events (MACCEs) and the impact of postoperative medications.

**Results:**

Of the patients, 20 172 underwent CABG. Surgery within 24 h of AMI diagnosis demonstrated the most favorable outcomes, reducing cardiac death, myocardial infarction recurrence, and target vessel revascularization. Delayed CABG within 3 days also outperformed surgery within 1–2 days post‐AMI. Additionally, postoperative aspirin use was associated with improved MACCE outcomes.

**Conclusion:**

CABG within 24 h of AMI diagnosis was associated with significantly minimized myocardial injury, emphasizing the critical role of rapid revascularization. Delayed CABG within 3 days related to better outcomes compared with that of surgery within 1–2 days. These findings provide evidence‐based recommendations for optimizing CABG timing in AMI patients, consequentially reducing morbidity and mortality.

## Graphical abstract

Performing surgery within 24 h of acute myocardial infarction (AMI) diagnosis minimizes myocardial damage. If surgery cannot occur within this timeframe, delaying it to after 3 days is preferable to 1–2 days, reducing risks such as cardiac death and unplanned revascularization. This study provides evidence‐based recommendations for optimizing coronary artery bypass grafting timing and improving postoperative care in AMI patients.

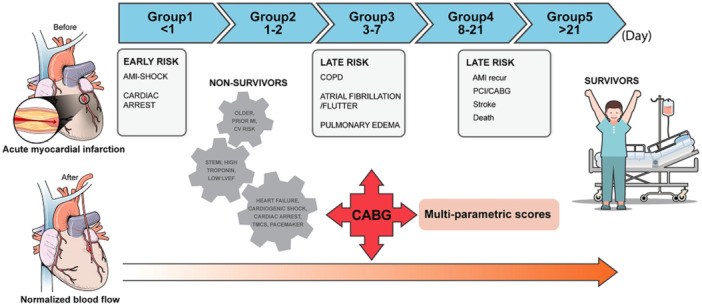

**Abbreviations**
ACS: acute coronary syndrome
AMI: acute myocardial infarction
ATT: average treatment effect among the treated
CABG: coronary artery bypass grafting
CVA: cerebrovascular
DAPT: dual antiplatelet therapy
HR: hazard ratio
LM: left main
MACCE: major adverse cardiac and cerebrovascular events
MI: myocardial infarction
NHIS: National Health Insurance Service
NSTEMI: non‐ST‐segment elevation myocardial infarction
PCI: percutaneous coronary intervention
STEMI: ST‐segment elevation myocardial infarction

## 1 Introduction

Acute myocardial infarction (AMI) is a global health concern, representing one of the leading causes of morbidity and mortality worldwide [1]. Although percutaneous coronary intervention (PCI) has become the main intervention method for AMI, coronary artery bypass grafting (CABG) is still a safe and feasible choice for patients with acute coronary syndrome (ACS). Moreover, it is an appropriate treatment for PCI failure, severe multivessel disease, or diabetes mellitus [2]. However, the optimal timing of CABG in patients with AMI is still a subject of ongoing research, with conflicting study results.

South Korea, with its robust national healthcare database, offers an invaluable resource for large‐scale studies that address this knowledge gap. The National Health Insurance Service (NHIS) of Korea maintains a comprehensive collection of patient medical histories, treatments, and outcomes. As such, a study using this database would be nationally representative and could provide globally applicable insights [3, 4].

In this study, we leveraged the robust national healthcare database in South Korea to address the knowledge gap regarding the optimal timing of CABG in patients with AMI. The study objectives were as follows: (1) utilize the Korean NHIS to identify a cohort of patients diagnosed with AMI who subsequently underwent CABG and categorize them based on the timing of their surgical intervention; (2) compare postoperative outcomes among these subgroups to elucidate the impact of surgical timing on clinical efficacy; and (3) assess the influence of postoperative medications on patient outcomes.

## 2 Materials and Methods

### 2.1 Study Population

We conducted a nationwide retrospective analysis using the National Health Claims database established by the NHIS of Korea. This database contains information regarding medical costs, precise details of prescribed medications, and medical history categorized by the International Classification of Diseases, Tenth Revision codes. The Korean population is mandated to subscribe to the NHIS rendering this cohort highly representative of the entire Korean population. Causes of death data were obtained from the National Institute of Statistics of Korea. Patients who underwent cardiac surgery were selected from the NHIS database.

Since 2007, the South Korean government has implemented measures to enhance the quality of cardiovascular disease treatment, including the “Financial Incentive to Quality Improvement Program for Myocardial Infarction.” Consequently, this study's cohort included patients with AMI spanning from January 2007 to December 2018, accounting for the wash‐out period. We identified 1 709 284 adults who had been diagnosed with AMI in this period.

Approval for this study was obtained from the Institutional Review Board of the Yonsei University Health System under approval number 4‐2020‐1392. The requirement for informed consent was waived due to the anonymization of personal information. All procedures adhered to the principles of the Declaration of Helsinki (2013 version).

### 2.2 Study Endpoints

The primary endpoint of this study aimed to identify a cohort of patients diagnosed with AMI from the broader Korean population, using data from the Health Insurance Review and Assessment Service. This cohort was further categorized based on those who underwent CABG, with a specific focus on comparing the time elapsed between diagnosis and surgery.

The secondary endpoint involved the assessment of major adverse cardiac and cerebrovascular events (MACCEs), encompassing cardiac death, cerebrovascular events (stroke), myocardial infarction (MI), and coronary artery reintervention, including reoperative CABG and PCI.

The tertiary endpoint examined clinical efficacy with respect to the post‐CABG medical treatment strategy, encompassing antiplatelet drugs, antithrombotic drugs, statins, and diabetes mellitus medication.

### 2.3 Statistical Analysis

Categorical variables are summarized as frequencies and percentages, and comparisons were made using Pearson's *χ* ^2^ test. Continuous variables were analyzed by calculating the mean and standard deviation.

For comparisons involving multiple treatment groups, the concept of average treatment among the treated (ATT) was employed. ATT was defined as a comparison of the mean outcome under a specific treatment (t′) as administered, with the mean outcome that would have occurred under another treatment (t″) among those initially treated with t′ [5]. Imbalances between groups for baseline comorbidities and medications were assessed using standardized mean differences and kernel density plots, with values > 0.2 indicating potential imbalances.

Cumulative incidence curves and the rates of secondary endpoint occurrence during the follow‐up period were generated using the Kaplan–Meier method. To control for multiple comparisons, Bonferroni's method was applied to adjust *p* values. The adjusted hazard ratio (HR) for each clinical endpoint was calculated using the Cox proportional hazards regression model. Significance was determined by a two‐sided *p* < 0.05. Statistical analyses were conducted using STATA Release 12 (STATA Corp, College Station, TX, USA) and R version 4.2.3 (The R Foundation).

## 3 Results

### 3.1 Incidence of AMI and CABG for AMI in South Korea

AMI was diagnosed based on criteria that included elevated total creatinine kinase or creatine kinase‐MB levels, exceedingly twice or thrice the upper limit of the normal range, elevated alternative cardiac markers, or the presence of AMI indicators as determined by electrocardiography or echocardiography.

In South Korea, we observed a distinctive trend in the overall incidence of AMI over the past decade, encompassing both ST‐segment elevation MI (STEMI) and non‐STEMI (NSTEMI) cases. This trend can be characterized by an M‐shaped curve. During our follow‐up period, STEMI cases showed fluctuations, while NSTEMI cases displayed a relatively stable incidence rate. A similar M‐shaped curve was also evident when examining CABG rates, as illustrated in Figure S1.

### 3.2 Study Population

To establish a specific study cohort, we applied eligibility criteria that excluded patients who had previously undergone CABG surgery (*n* = 206) and nonadult patients under the age of 18 (*n* = 918). This resulted in an inclusion of adult patients diagnosed with AMI between 2007 and 2018, who had not received prior CABG surgery (*n* = 1 705 843).

**Figure 1:**
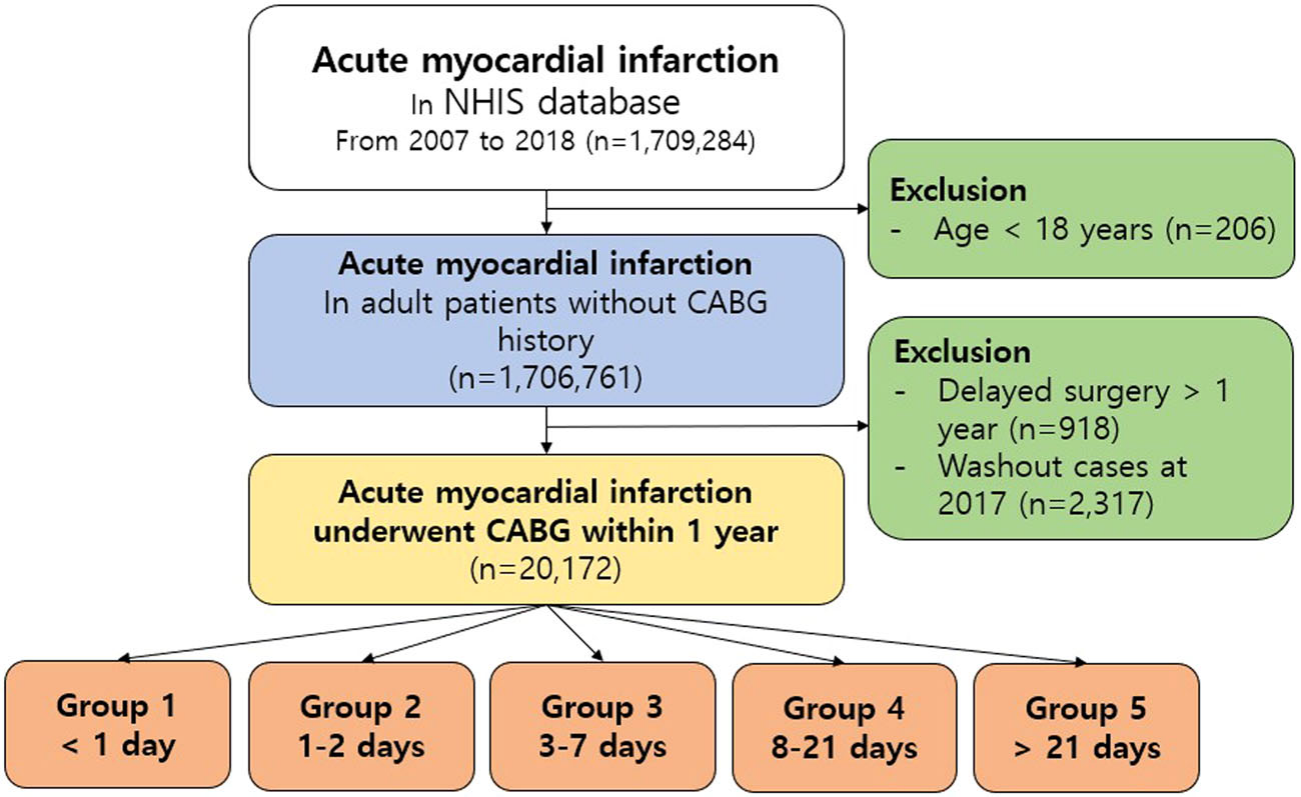
Summary flow diagram of the study population. CABG, coronary artery bypass grafting; NHIS, National Health Insurance Service.

Patients who did not undergo CABG within 1 year of their AMI diagnosis were further excluded. The final cohort comprised adult patients (aged ≥ 18 years) who had received CABG within a year of their AMI diagnosis (*n* = 20 172). The patients were categorized into five subgroups based on the timing of their surgery (Figure 1).

### 3.3 Baseline Demographic, Clinical, and Medical Characteristics After ATT Weighting

Supporting Information S1: Table S1 displays the baseline demographic, clinical, and medical characteristics following ATT (inverse probability treatment weighting) adjustment. The mean age of the entire study population at the time of surgery was 71.2 ± 9.5 years, with a predominantly male patient cohort.

### 3.4 Clinical Outcomes in Terms of MACCEs

The median duration of follow‐up was 3.8 ± 4.1 years. Table 1 provides an overview of the clinical outcomes among the patients studied over time, including cardiac‐related deaths (6.92%, group 1 vs. 13.00%, group 2; risk difference: 6.08%), the recurrence rate of MI (23.77%, group 1 vs. 46.44%, group 3; risk difference: 22.67%), and target vessel revascularization via PCI (8.74%, group 1 vs. 3.95% group 3; risk difference: 4.79%).

**Table 1:** Risks of MACCEs and individual treatment effects after propensity score matching.

	Total (*N* = 20 172)	< 1 day (*n* = 16 322)	1–2 days (*n* = 585)	3–7 days (*n* = 792)	8–21 days (*n* = 1068)	> 21 days (*n* = 1405)
MACCEs (%)	11 844 (58.72)	9337 (57.20)	395 (67.52)	506 (63.89)	673 (63.01)	933 (66.41)
Duration from CABG (median, IQR, days)	66 (30, 539)	56 (30, 535)	117 (40, 498)	126 (33, 596)	120 (35, 539)	113 (31, 572)
Cardiac death (%)	1741 (8.63)	1129 (6.92)	76 (13.00)	130 (16.41)	143 (13.39)	263 (18.72)
Duration from CABG (median, IQR, days)	45 (12, 635)	40 (11, 631)	103 (20, 386)	231.5 (16, 1432)	103 (20, 718)	55 (16, 465)
Recurrent MI (%)	3229 (27.26)	2219 (23.77)	180 (45.57)	235 (46.44)	275 (40.86)	320 (34.30)
Duration from CABG (median, IQR, days)	48 (35, 209)	39 (33, 78)	118 (50, 404)	160 (50, 596)	163 (50, 435)	233.5 (58, 687)
TVR (PCI+CABG)	1003 (8.47)	851 (9.11)	29 (7.34)	21 (4.15)	51 (7.58)	51 (5.47)
Duration from CABG (median, IQR, days)	420 (120, 1077)	418 (117, 1051)	369 (127, 1104)	540 (148, 1177)	442 (136, 1360)	492 (194, 1061)
PCI (%)	960 (8.11)	816 (8.74)	29 (7.34)	20 (3.95)	48 (7.13)	47 (5.04)
Duration from CABG (median, IQR, days)	428 (130, 1107)	425 (125, 1070)	369 (127, 1104)	480 (137, 1238)	455 (146, 1378)	602 (210, 1061)
CABG (%)	50 (0.42)	42 (0.45)	0 (0.00)	1 (0.20)	3 (0.45)	4 (0.43)
Duration from CABG (median, IQR, days)	72.5 (30, 577)	72.5 (30, 552)	—	789	38 (4, 668)	222 (3, 960)
CVA (stroke) events (%)	6212 (52.45)	499 (53.53)	173 (43.80)	224 (44.27)	323 (47.99)	494 (52.95)
Duration from CABG (median, IQR, days)	73 (27, 648)	77 (28, 684)	85 (28, 609)	50 (24, 436)	57 (23, 582)	65.5 (25, 458)

Abbreviations: CABG, coronary artery bypass grafting; CVA, cerebrovascular events; IQR; interquartile range; MACCEs, major adverse cardiac and cerebrovascular events; MI, myocardial infarction; PCI, percutaneous coronary intervention; TVR, target vessel revascularization.

### 3.5 Relationship Between Surgical Timing for AMI and MACCEs

**Figure 2:**
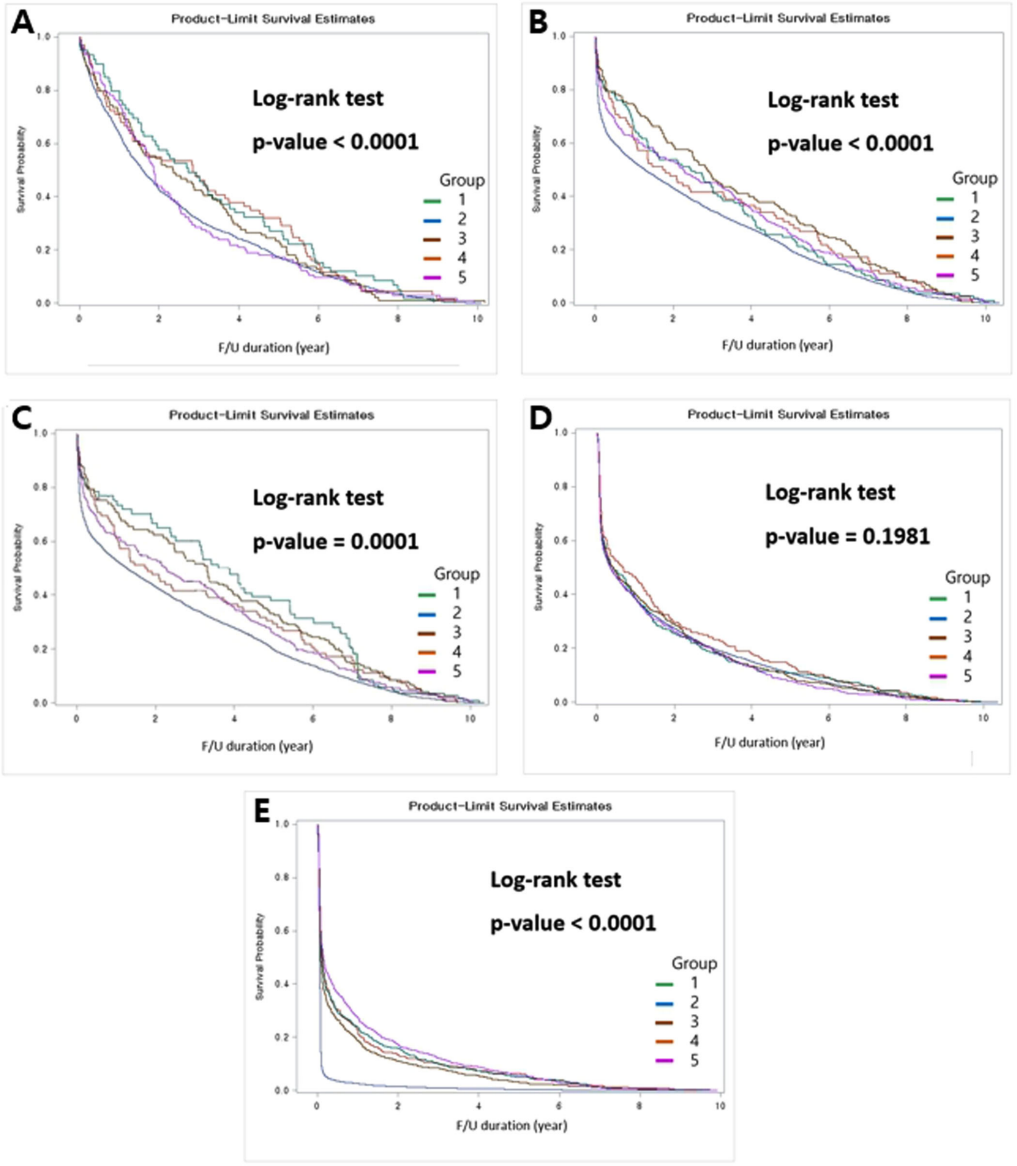
Kaplan–Meier plots. (A) Freedom from cardiac death, (B) freedom from recurrent myocardial infarction, (C) freedom from target vessel revascularization, (D) freedom from stroke, (E) freedom from major adverse cardiac and cerebrovascular events.

In terms of cardiac‐related deaths, both groups 2 and 5 displayed unfavorable outcomes (*p* < 0.0001) (Figure 2A and Table 2). Furthermore, group 2 distinctly exhibited less favorable outcomes in comparison to that of the other groups concerning recurrent MI after surgery (Figure 2B and Table 2). The outcomes of target vessel revascularization (PCI+CABG) followed a similar pattern, with group 2 displaying the poorest results, and group 1 showing the most favorable outcomes (Figure 2C and Table 2). There were no statistically significant differences in the incidence of strokes among the groups (*p* = 0.198) (Figure 2D and Table 2). Finally, Kaplan–Meier analysis unveiled that group 2 (CABG 1–2 days after AMI diagnosis) exhibited the least favorable MACCE outcomes overall (log‐rank test, *p* < 0.0001). Intergroup comparisons solidified the significant difference between group 2 and the remaining groups (Figure 2E and Table 2).

**Table 2:** Multiple comparison adjustment for the Log‐rank test.

Intergroup comparison	MACCEs		Cardiac death		MI		TVR		CVA	
	Raw^†^	Bonferroni^‡^	Raw^†^	Bonferroni^‡^	Raw^†^	Bonferroni^‡^	Raw^†^	Bonferroni^‡^	Raw^†^	Bonferroni^‡^
Overall	< 0.0001		< 0.0001		< 0.0001		0.0001		0.1981	
1 vs. 2	< 0.0001	< 0.0001	< 0.0001	< 0.0001	0.0571	0.5713	< 0.0001	< 0.0001	0.7243	> 0.9999
1 vs. 3	0.0181	0.1813	0.0677	0.6772	< 0.0001	< 0.0001	0.0169	0.1694	0.4478	> 0.9999
1 vs. 4	0.8010	> 0.999	0.9981	> 0.9999	0.1900	> 0.9999	< 0.0001	< 0.0001	0.8114	> 0.9999
1 vs. 5	0.0169	0.1694	< 0.0001	< 0.0001	0.1713	> 0.9999	< 0.0001	< 0.0001	0.8632	> 0.9999
2 vs. 3	< 0.0001	< 0.0001	< 0.0001	< 0.0001	< 0.0001	< 0.0001	< 0.0001	< 0.0001	0.9993	> 0.9999
2 vs. 4	< 0.0001	< 0.0001	< 0.0001	< 0.0001	< 0.0001	< 0.0001	< 0.0001	< 0.0001	0.9921	> 0.9999
2 vs. 5	< 0.0001	< 0.0001	0.5781	> 0.9999	0.0018	0.0182	0.0181	0.1813	0.9997	> 0.9999
3 vs. 4	0.2497	> 0.9999	0.0151	0.1517	< 0.0001	< 0.0001	0.5010	> 0.9999	0.9999	> 0.9999
3 vs. 5	< 0.0001	< 0.0001	< 0.0001	< 0.0001	< 0.0001	< 0.0001	< 0.0001	< 0.0001	0.9991	> 0.9999
4 vs. 5	0.0252	0.2521	< 0.0001	< 0.0001	0.2190	> 0.9999	0.0252	0.2521	0.0487	0.4876

*Note:* Bonferroni's^‡^ method: adjusted *p* value = raw *p* value^†^ × comparison frequency (_5_C_2_ = 10).

Abbreviatons: CVA, cerebrovascular events; MACCEs, major adverse cardiac and cerebrovascular events; MI, myocardial infarction; TVR, target vessel revascularization.

### 3.6 Correlation Between MACCEs After AMI and Surgical Timing

Following ATT weighting, the Cox regression multivariable analysis revealed that age (*p* < 0.0001), diabetes mellitus (*p* < 0.0001), chronic renal failure (*p* < 0.0001), heart failure, and ischemic heart disease (*p* < 0.0001) emerged as risk factors for MACCEs (Table 3). Additionally, the timing of surgical intervention after AMI was identified as an independent risk factor for all MACCEs. Specifically, CABG surgery performed within 1–2 days after AMI diagnosis was associated with a significant risk increase. Notably, group 1 exhibited more favorable outcomes in comparison to that of the other groups. Among the individual components of MACCEs, target vessel revascularization revealed less and more favorable results in groups 2 and 1, respectively.

**Table 3:** Multivariate Cox regression analysis for endpoints after CABG for AMI.

Variables	MACCEs		Cardiac death		MI		TVR		CVA	
	Adjusted HR (95% CI)	*p* values	Adjusted HR (95% CI)	*p* values	Adjusted HR (95% CI)	*p* values	Adjusted HR (95% CI)	*p* values	Adjusted HR (95% CI)	*p* values
Age at CABG	1.013 (1.011–1.015)	< 0.0001	1.053 (1.049–1.057)	< 0.0001	1.000 (0.997–1.003)	0.9935	1.026 (1.024–1.029)	< 0.0001	0.985 (0.981–0.990)	< 0.0001
Sex										
Male	1 (ref)		1 (ref)		1 (ref)		1 (ref)		1 (ref)	
Female	0.968 (0.929–1.009)	0.1202	1.007 (0.937–1.082)	0.8563	1.100 (1.025–1.182)	0.0086	0.928 (0.881–0.978)	0.0054	1.101 (0.987–1.230)	0.0855
Underlying disease										
Hypertension	1.064 (0.996–1.137)	0.0673	1.048 (0.922–1.191)	0.4756	0.920 (0.826–1.024)	0.1250	1.334 (1.217–1.462)	< 0.0001	1.219 (1.023–1.451)	0.0266
Diabetes mellitus	1.200 (1.150–1.252)	< 0.0001	1.275 (1.176–1.381)	< 0.0001	1.030 (0.959–1.106)	0.4188	1.409 (1.333–1.489)	< 0.0001	1.164 (1.042–1.301)	0.0074
Chronic renal failure	1.276 (1.208–1.349)	< 0.0001	2.466 (2.266–2.683)	< 0.0001	1.092 (0.985–1.210)	0.0948	1.370 (1.276–1.470)	< 0.0001	2.018 (1.761–2.312)	< 0.0001
Heart failure	1.090 (1.045–1.137)	< 0.0001	1.430 (1.329–1.539)	< 0.0001	1.036 (0.963–1.115)	0.3410	1.022 (0.968–1.078)	0.4393	1.074 (0.957–1.205)	0.2254
Ischemic heart disease	0.873 (0.823–0.926)	< 0.0001	0.662 (0.599–0.731)	< 0.0001	0.770 (0.694–0.854)	< 0.0001	1.155 (1.067–1.251)	0.0004	1.078 (0.915–1.269)	0.3692
PCI history	1.062 (1.018–1.108)	0.0058	1.191 (1.103–1.286)	< 0.0001	—	—	—	—	—	—
Stoke history	2.128 (2.049–2.210)	< 0.0001	1.156 (1.078–1.240)	< 0.0001	—	—	—	—	—	—
Postoperative medicine (time‐varying variable)										
Aspirin	0.347 (0.327–0.368)	< 0.0001	0.604 (0.526–0.694)	< 0.0001	0.404 (0.366–0.447)	< 0.0001	0.493 (0.454–0.536)	< 0.0001	0.713 (0.589–0.863)	0.0005
Anticoagulant	0.630 (0.598–0.664)	< 0.0001	1.457 (1.279–1.661)	< 0.0001	0.571 (0.523–0.623)	< 0.0001	0.839 (0.780–0.902)	< 0.0001	1.319 (1.113–1.563)	0.0014
β‐blocker	0.935 (0.893–0.979)	0.0042	1.016 (0.925–1.117)	0.7352	0.971 (0.894–1.054)	0.4848	1.101 (1.035–1.171)	0.0023	1.050 (0.930–1.187)	0.4287
Statin	0.809 (0.771–0.849)	< 0.0001	0.750 (0.682–0.824)	< 0.0001	0.911 (0.835–0.994)	0.0372	0.902 (0.846–0.962)	0.0018	1.040 (0.910–1.190)	0.5619
Duration from AMI to CABG										
< 1 day	0.776 (0.702–0.859)	< 0.0001	0.759 (0.691–0.970)	0.0405	0.758 (0.649–0.887)	0.0005	0.640 (0.453–0.903)	0.0111	0.948 (0.797–1.126)	0.5410
1–2 days	1 (ref)		1 (ref)		1 (ref)		1 (ref)		1 (ref)	
3–7 days	0.935 (0.820–1.067)	0.3165	0.990 (0.746–1.313)	0.9453	0.344 (0.301–0.394)	< 0.0001	0.676 (0.500–0.912)	0.0105	0.965 (0.820–1.134)	0.6627
8–21 days	0.859 (0.758–0.972)	0.0163	0.751 (0.726–1.244)	0.0121	0.523 (0.779–1.094)	0.0354	0.760 (0.595–0.969)	0.0271	0.907 (0.794–1.036)	0.1519
> 21 days	0.840 (0.746–0.945)	0.0038	1.217 (0.950–1.559)	0.1207	0.772 (0.655–0.910)	0.0020	0.752 (0.552–1.024)	0.0111	1.053 (0.903–1.228)	0.5088

Abbreviations: AMI, acute myocardial infarction; CABG, coronary artery bypass grafting; IQR, interquartile range; MACCEs, major adverse cardiac and cerebrovascular event; MI, myocardial infarction; PCI, percutaneous coronary intervention; TVR, target vessel revascularization.

### 3.7 Impact of Postoperative Long‐Term Medication With Aspirin

Through a logistic regression model adjusted for baseline characteristics, we investigated the influence of medications, including antiplatelet drugs, antithrombotic drugs, statins, and diabetes mellitus medications on postoperative outcomes. Improved MACCE outcomes were associated with aspirin, anticoagulants, beta‐blockers, and statins across the entire patient population. However, there was no significant difference in MACCE outcomes between patients who had taken aspirin for more and less than 6 months (Supporting Infomation S1: Table S2).

## 4 Discussion

This nationwide study found that only 1.18% of patients diagnosed with AMI received surgical treatment. Through ATT estimation, we found that timely reperfusion within 24 h of the initial diagnosis was most effective in patients with MI. This finding indicates the critical importance of timely revascularization in mitigating the extent of myocardial injury and preserving cardiac function. Furthermore, surgical treatment between 1 and 2 days posed the highest risk (Graphical abstract). This may be due to the systemic inflammatory response caused by MI and the fact that this timeframe is most affected during shock [6, 7, 8, 9]. This report offers a comprehensive understanding of how the duration of ischemia affects both the size of the myocardial infarct and the extent of damage, emphasizing the importance of timely medical intervention.

Studies report variable results regarding the optimum time for CABG after MI and the effect on mortality rate [8, 10, 11, 12, 13]. Notably, most studies are single‐center reports that do not include patients who underwent CABG within 24 h of AMI. However, this study examined the effects of the full‐time period using the Korean national database. We found the golden time for patients undergoing CABG for MI was within a day. Furthermore, in instances where this was not feasible, a waiting period extending up to 3 days post‐diagnosis was associated with reduced postoperative MACCE outcomes. Due to the progress of myocardial protection and mechanical support technology and the improvement of anesthesia and perioperative management in patients undergoing cardiac surgery, surgical intervention could play an important role in the early stages of treatment for patients with AMI.

A 2022 joint ESC/EACTS review of the 2018 guidelines regarding LM revascularization suggested that the procedure was for stable patients with a coronary anatomy suitable for surgical revascularization and low predicted surgical mortality. According to the guidelines, surgical risk is predicted by the Society of Thoracic Surgeons score plus additional factors not accounted for by the risk score [2]. We suggest incorporating a preoperative risk factor into the new guidelines, including the time interval from AMI diagnosis to CABG surgery.

For patients with STEMI, previous guidelines recommend delaying CABG for 3–7 days or until patients are stabilized while acknowledging that it can be performed within 48 h for patients with multivessel coronary artery disease with recurrent angina [14, 15]. Although this study included fewer patients with STEMI than those with NSTEMI, patients with NSTEMI are often relatively stable. It is likely that patients with STEMI influenced the characteristics of this study population. Further research is required to assess patients according to MI type, whether STEMI or NSTEMI.

The perioperative use of aspirin has been associated with a significant reduction in 30‐day mortality without significant bleeding complications [16]. In our study, aspirin use reduced the incidence of MACCEs and individual outcome components (Table 3). However, guidelines and clinical practices lack uniformity and specificity regarding dual antiplatelet therapy for patients undergoing coronary bypass grafting, especially in the context of chronic coronary syndrome [17]. The success of CABG depends mainly on the patency of graft vessels, which is closely related to long‐term survival after CABG [18]. The 2017 ESC guidelines recommend the use of dual antiplatelet therapy (DAPT) a year after CABG in patients with acute coronary syndrome (ACS), although the available evidence is limited [19]. Thus, the appropriate choice between aspirin and P2Y12 inhibitors for CABG remains unclear. In this study, there was no significant difference in MACCE between patients who had taken aspirin for more or less than 6 months. The latest review of DAPT and CABG, with a focus on ACS, supports the use of DAPT with aspirin and ticagrelor in patients with ACS after CABG [20]. Additional medical therapy strategies are necessary to improve long‐term graft patency after CABG.

The advantage of ATT is that it specifically estimates treatment effects, allowing for the evaluation of the real‐world impact of a particular treatment or intervention. ATT also provides insights that can be immediately useful for policy decisions or clinical guidelines that concern the treated population. This is especially relevant when treatment assignment is nonrandom, and systematic differences exist between treated and untreated groups.

### 4.1 Study Limitations

The study had limitations. First, we did not implement random allocation between the different surgical methods. Furthermore, the main limitation of this study was that we did not perform an AMI subtype (STEMI or NSTEMI) analysis. However, we aimed to reach a general conclusion using national data and targeting a large patient population. Specialized statistical techniques were employed to control for imbalances across multiple groups. Second, because the NHIS only contains information about claims data, the reasons for determining the timing of CABG could not be determined. Third, there was no subgroup analysis of STEMI or NSTEMI. Finally, owing to the nature of the retrospective cohort study based on claims, the results of this study cannot be used to prove causal relationships.

## 5 Conclusion

In conclusion, performing surgical intervention within 24 h of diagnosing an acute myocardial infarction (AMI) is effective in minimizing myocardial damage. However, if surgery cannot be performed within 24 h, it is better to perform it after 3 days rather than within 1–2 days, as this approach is more effective in reducing the risks of cardiac death, nonfatal myocardial infarction, and unplanned revascularization. In this study, using the ATT estimation provides a more focused evaluation of the treatment program's efficacy for the subset of treated patients, offering detailed insights. Therefore, this study presents evidence‐based recommendations for optimizing the timing of CABG in AMI patients and refining postoperative treatment guidelines to improve patient care and reduce AMI‐related morbidity and mortality.

## Ethics Statement

Approval for this study was obtained from the Institutional Review Board of the Yonsei University Health System under approval number 4‐2020‐1392. All procedures adhered to the principles of the Declaration of Helsinki (2013 version).

## Consent

The requirement for informed consent was waived due to the anonymization of personal information.

## Conflicts of Interest

The authors declare no conflicts of interest.

## Supporting information

Supporting information Figure S1. (A) The incidence rate of total acute myocardial infarction (STEMI and NSTEMI and total AMI, respectively) in South Korea between 2008 and 2018. (B) Annual trends of CABG surgery in South Korea between 2008 and 2018.

Supporting information.

Supporting information.

## Data Availability

Data sharing is not applicable to this article as no datasets were generated or analyzed during the current study.
