# Supplementary material for: Optimal Revascularization Timing of Coronary Artery Bypass Grafting in Acute Myocardial Infarction
Source: Clin Cardiol. 2024 Aug 14;47(8):e24325. doi: 10.1002/clc.24325 (PMC11322592; doi:10.1002/clc.24325)
Supplement: Supplementary file 2 — Supporting information. [file CLC-47-e24325-s001.docx]

**Supporting information Figure S1**. (A) The incidence rate of total acute myocardial infarction (STEMI and NSTEMI, and total AMI, respectively) in South Korea between 2008 and 2018. (B) Annual trends of CABG surgery in South Korea between 2008 and 2018.
