# Supplementary material for: Optimal Revascularization Timing of Coronary Artery Bypass Grafting in Acute Myocardial Infarction
Source: Clin Cardiol. 2024 Aug 14;47(8):e24325. doi: 10.1002/clc.24325 (PMC11322592; doi:10.1002/clc.24325)
Supplement: Supplementary file 3 — Supporting information. [file CLC-47-e24325-s003.docx]

**Supplementary Table S1. Baseline characteristics and medications and ATT of CABG timing after AMI in pairwise comparisons using propensity score matching**

| **Variables** | **<1 day**  **(n=16,322)** | **1–2 days**  **(n=585)** | | | **3–7 days**  **(n=792)** | | | **8–21 days**  **(n=1,068)** | | | **>21 days**  **(n=1,405)** | | |
| --- | --- | --- | --- | --- | --- | --- | --- | --- | --- | --- | --- | --- | --- |
|  | **Control (%)** | **ATT Weighted** | **ATT**  **(% point)** | **p-value** | **Treatment**  **(%)** | **ATT**  **(% point)** | **p-value** | **Treatment (%)** | **ATT**  **(% point)** | **p-value** | **Treatment**  **(%)** | **ATT**  **(% point)** | **p-value** |
| Age, y | 68.9±10.2 | 70.6±9.7 |  |  | 72.2±11.5 |  |  | 72.8±12.1 |  |  | 72.5±9.9 |  |  |
| Women | 4,401  (26.96) | 157  (26.84) | -0.12 | 0.406 | 220  (27.78) | 0.82 | 0.277 | 289  (27.06) | 0.10 | 0.487 | 383  (27.26) | 0.30 | 0.374 |
| Comorbidity | | | | | | | | | | | | | |
| Hypertension | 12,470  (76.40) | 458  (78.29) | 1.89 | 0.091 | 612  (77.27) | 0.87 | 0.260 | 806  (75.47) | -0.93 | 0.194 | 1083  (77.08) | 0.68 | 0.319 |
| CKD | 1240  (7.60) | 42  (7.18) | -0.42 | 0.358 | 59  (7.45) | -0.15 | 0.389 | 88  (8.24) | 0.64 | 0.321 | 111  (7.97) | 0.37 | 0.367 |
| Diabetes mellitus | 7,181  (44.00) | 259  (44.27) | 0.27 | 0.371 | 354  (44.69) | 0.69 | 0.320 | 475  (44.48) | 0.48 | 0.353 | 622  (44.27) | 0.27 | 0.371 |
| Atherosclerotic cardiovascular disease | | | | | | | | | | | | | |
| Prior MI | 2219  (13.60) | 73  (12.48) | -1.12 | 0.145 | 102  (12.88) | -0.72 | 0.322 | 128  (11.96) | -1.64 | 0.114 | 189  (13.45) | -0.15 | 0.389 |
| Prior PCI | 328  (2.01) | 13  (2.22) | 0.21 | 0.382 | 18  (2.27) | 0.26 | 0.373 | 22  (2.06) | 0.05 | 0.749 | 38  (2.70) | 0.69 | 0.320 |
| Prior ischemic stroke | 2807  (17.20) | 106  (18.12) | 0.92 | 0.143 | 136  (17.17) | -0.03 | 0.757 | 183  (17.13) | -0.07 | 0.511 | 264  (18.79) | 1.59 | 0.127 |
| PAOD | 2660  (16.30) | 106  (18.12) | 1.82 | 0.098* | 135  (17.05) | 0.75 | 0.316 | 187  (17.51) | 1.21 | 0.138 | 225  (16.01) | -0.29 | 0.373 |
| Medication for cardiovascular system | | | | | | | | | | | | | |
| Aspirin | 10739  (65.79) | 382  (65.30) | -0.49 | 0.354 | 506  (63.89) | -1.90 | 0.087 | 695  (65.07) | -0.72 | 0.322 | 920  (65.48) | -0.31 | 0.373 |
| Clopidogrel | 7605  (46.59) | 274  (46.84) | 0.25 | 0.375 | 374  (47.22) | 0.63 | 0.326 | 495  (46.35) | -0.24 | 0.376 | 666  (47.40) | 0.81 | 0.286 |
| β-blocker | 8471  (51.90) | 299  (51.11) | -0.79 | 0.301 | 415  (52.40) | 0.50 | 0.352 | 550  (51.50) | -0.40 | 0.361 | 738  (52.53) | 0.63 | 0.326 |
| RAAS blockade | 6085  (37.28) | 220  (37.61) | 0.33 | 0.370 | 297  (37.50) | 0.22 | 0.380 | 402  (37.64) | 0.36 | 0.378 | 514  (36.58) | -0.7 | 0.321 |
| Year of CABG |  | 0.195 | | | 0.101 | | | 0.130 | | | 0.051 | | |
| 2007-2010 | 5,325  (32.62) | 189  (32.31) | -0.31 |  | 262  (33.08) | 0.46 |  | 357  (33.43) | 0.81 |  | 487  (34.66) | 2.04 |  |
| 2011-2014 | 4,451  (27.27) | 165  (28.21) | 0.94 |  | 226  (28.54) | 1.27 |  | 299  (28.00) | 0.73 |  | 393  (27.97) | 0.70 |  |
| 2015-2018 | 6,546  (40.11) | 231  (39.49) | -0.62 |  | 304  (38.38) | -1.73 |  | 412  (38.58) | -1.53 |  | 525  (37.40) | -2.71 |  |

*AMI; acute myocardial infarction, ATT; average treatment effects, CABG; coronary artery bypass grafting, CKD; chronic kidney disease, MI; myocardial infarction, PAOD; peripheral arterial occlusive disease, PCI; percutaneous coronary intervention, RAAS; renin-angiotensin-aldosterone system

**Supplementary Table S2. Multivariate Cox regression analysis for postoperative drug effect**

| Variables | **MACCEs** | | **Cardiac death** | | **MI** | | **TVR** | | **CVA** | |
| --- | --- | --- | --- | --- | --- | --- | --- | --- | --- | --- |
|  | Adjusted HR  (95% CI) | p-values | Adjusted HR  (95% CI) | p-values | Adjusted HR  (95% CI) | p-values | Adjusted HR  (95% CI) | p-values | Adjusted HR  (95% CI) | p-values |
| Postoperative medicine (time-varying variable) | | | | | | | | | | |
| Aspirin | 0.347  (0.327-0.368) | <.0001 | 0.604  (0.526-0.694) | <.0001 | 0.404  (0.366-0.447) | <.0001 | 0.493  (0.454-0.536) | <.0001 | 0.713  (0.589-0.863) | 0.0005 |
| Anticoagulant | 0.630  (0.598-0.664) | <.0001 | 1.457  (1.279-1.661) | <.0001 | 0.571  (0.523-0.623) | <.0001 | 0.839  (0.780-0.902) | <.0001 | 1.319  (1.113-1.563) | 0.0014 |
| β -blocker | 0.935  (0.893-0.979) | 0.0042 | 1.016  (0.925-1.117) | 0.7352 | 0.971  (0.894-1.054) | 0.4848 | 1.101  (1.035-1.171) | 0.0023 | 1.050  (0.930-1.187) | 0.4287 |
| Statin | 0.809  (0.771-0.849) | <.0001 | 0.750  (0.682-0.824) | <.0001 | 0.911  (0.835-0.994) | 0.0372 | 0.902  (0.846-0.962) | 0.0018 | 1.040 (0.910-1.190) | 0.5619 |
| Aspirin medication duration after CABG | | | | | | | | | | |
| < 180, days | 1 (ref) |  | 1 (ref) |  | 1 (ref) |  | 1 (ref) |  | 1 (ref) |  |
| 180~300, days | 1.155  (0.983-1.358) | 0.0797 | 1.225  (1.042-1.440) | 0.0141 | 1.062  (0.796-1.415) | 0.6841 | 1.081  (0.810-1.442) | 0.5970 | 1.247  (0.863-1.782) | 0.6993 |
| >300, days | 1.129  (0.993-1.283) | 0.0650 | 1.235  (1.086-1.405) | 0.0013 | 1.089  (0.868-1.366) | 0.4618 | 1.134  (0.904-1.424) | 0.2775 | 1.371  (0.977-1.264) | 0.3725 |

*CABG; coronary artery bypass grafting, CVA; cerebrovascular events, CI; confidence interval, HR; hazard ratio, MACCEs; major adverse cardiac and cerebrovascular event, MI; myocardial infarction, TVR; target vessel revascularization
